# Lived experiences and acceptability challenges among caregivers of people with epilepsy in Limpopo and Mpumalanga, South Africa

**DOI:** 10.3389/fpsyg.2026.1857742

**Published:** 2026-06-17

**Authors:** Happiness Ngobeni, Thendo Gertie Makhado, Lufuno Makhado

**Affiliations:** 1 Department of Public Health, Faculty of Health Sciences, University of Venda, Thohoyandou, South Africa; 2 Department of Advanced Nursing Sciences, Faculty of Health Sciences, University of Venda, Thohoyandou, South Africa

**Keywords:** barriers, discrimination, epilepsy, facilitators, people with epilepsy, seizure disorder, social acceptance, stigma

## Abstract

**Background:**

Epilepsy places substantial demands on family caregivers, particularly in rural settings where stigma, poverty, limited access to health services, and misinformation shape care. In South Africa, little qualitative evidence has explored how caregivers experience epilepsy care across rural provinces and how social acceptability influences caregiving.

**Objective:**

To explore the lived experiences and acceptability challenges among caregivers of people with epilepsy (PWE) in Limpopo and Mpumalanga Provinces, South Africa.

**Methods:**

A qualitative exploratory-descriptive study was conducted among informal caregivers of PWE in rural and peri-urban communities across both provinces. Data were collected through in-depth semi-structured interviews and analyzed inductively using thematic analysis.

**Results:**

Five interrelated themes captured caregivers’ experiences: (1) caregiving as constant vigilance, (2) emotional and physical strain in responding to unpredictable seizures, (3) stigma and low social acceptability of epilepsy, (4) caregiving under structural and service constraints, and (5) coping, endurance, and unmet support needs. Caregivers described continuous supervision, fear during seizures, physical exhaustion, and major disruption to work, mobility, and household routines. Their experiences were further shaped by community judgment, misconceptions about epilepsy, and exclusion in schools, churches, and social spaces. Poverty, transport barriers, limited practical support, and inadequate caregiver guidance from services intensified these burdens. Although caregivers demonstrated endurance through treatment monitoring, family responsibility, faith, and selective use of available support, these coping mechanisms were often fragile and insufficient.

**Conclusion:**

Caregiving for PWE in rural South Africa is a demanding and socially shaped experience that extends beyond seizure management to include emotional labor, social negotiation, and structural hardship. Interventions to improve epilepsy outcomes should include caregiver education, stigma reduction, family-centered psychosocial support, and stronger community- and primary healthcare-based support systems.

## 1 Introduction

Epilepsy is one of the most common neurological disorders globally and remains a major public health concern, particularly in low- and middle-income countries where access to diagnosis, treatment, and long-term support is often uneven. In South Africa, epilepsy is associated with recurring seizures, disrupted schooling and employment, treatment challenges, and reduced social participation (Makhado et al., 2024; Musekwa et al., 2024; WHO, 2024). Although epilepsy is experienced by the individual, its effects extend to family members who provide daily care and support. In settings where health and social services are limited, informal caregivers often assume responsibility for treatment monitoring, seizure safety, daily supervision, and responding to the social consequences of epilepsy (Pokharel et al., 2021; Yu et al., 2022).

Caregiving for people with epilepsy (PWE) can be demanding and complex. Previous studies have shown that caregivers often experience fear of sudden seizures, emotional distress, social stigma, and difficulty balancing caregiving with employment, education, and other household responsibilities (Keikelame and Swartz, 2016; Yu et al., 2022). In African settings, these challenges may be intensified by limited access to epilepsy-specific information, treatment gaps, and community beliefs that frame epilepsy in terms of witchcraft, possession, curses, or contagion rather than as a medical condition (Kaddumukasa et al., 2018; Nemathaga et al., 2023). Such beliefs can shape help-seeking behavior, delay access to formal care, and expose both PWE and their families to judgment, discrimination, and social exclusion (Agbetou et al., 2023; Kaddumukasa et al., 2018).

These issues are especially relevant in rural South African provinces such as Limpopo and Mpumalanga, where epilepsy care is often delivered in contexts marked by poverty, long travel distances to health facilities, limited specialist services, and uneven access to health information (Ngene et al., 2023; Wagner et al., 2020). Research from these provinces has shown that PWE and their caregivers frequently face misconceptions about the condition, inadequate guidance on seizure management, and gaps in provider support (Makhado et al., 2024; Musekwa et al., 2023). Health care providers themselves have reported limitations in epilepsy-related knowledge and counseling capacity, which may reduce the quality of support available to caregivers in primary and community care settings (Musekwa et al., 2024). In such environments, caregivers are often left to manage the practical demands of seizures and also the emotional strain, financial consequences, and social misunderstandings that surround epilepsy.

Despite growing literature on epilepsy-related knowledge, attitudes, practices, and barriers to care, fewer studies have examined how caregivers in rural South African settings experience caregiving in everyday life. Existing work has often focused on caregiver burden in general terms or on knowledge and practices, with less attention to how caregivers interpret their roles, navigate stigma and social acceptability, cope with uncertainty, and experience the wider structural conditions that shape care’ (Bagherian et al., 2021; Musekwa and Makhado, 2023). A richer qualitative understanding of caregivers’ lived experiences is important because it can illuminate how caregiving is organized in daily practice, how families manage risk and uncertainty, and how social and health-system contexts influence both caregiver well-being and the care received by PWE. Because epilepsy caregiving in rural settings is shaped by clinical uncertainty, social stigma, family responsibility, and limited service support, this study drew on stress, coping, and adaptation frameworks to interpret caregivers’ experiences within their social and structural context.

This study was informed by the transactional stress and coping model and the caregiving stress process model. The transactional stress and coping model conceptualizes stress as a dynamic relationship between individuals and their environment, shaped by how people appraise demands and the coping resources available to them (Lazarus and Folkman, 1984). In caregiving research, the stress process model further explains how care-related demands, role strains, social support, coping resources, and contextual conditions interact to shape caregiver burden and well-being (Pearlin et al., 1990). These frameworks are relevant to epilepsy caregiving because caregivers often experience seizure unpredictability, fear of injury, emotional distress, social stigma, financial strain, and disruption to work and family life (Hansen et al., 2018; Keikelame and Swartz, 2016; Sabo et al., 2020; Yu et al., 2022).

A psychosocial adaptation perspective was also used to interpret how caregivers adjust emotionally, socially, and practically to the demands of caring for PWE. Psychosocial adaptation is particularly relevant in rural and low-resource settings, where caregiving is shaped not only by the clinical condition but also by poverty, limited access to health services, cultural beliefs, stigma, and weak formal support systems (Kaddumukasa et al., 2018; Makhado et al., 2024; Ngene et al., 2023; Wagner et al., 2020). Together, these frameworks guided interpretation of the findings by moving the analysis beyond description toward understanding how caregivers appraise caregiving demands, mobilize coping strategies, and adapt under conditions of social and structural constraint.

The aim of this study was to explore the lived experiences and challenges of acceptability faced by informal caregivers of PWE in rural and peri-urban communities in Limpopo and Mpumalanga Provinces, South Africa. Specifically, the study sought to understand how caregivers experience daily caregiving responsibilities, negotiate stigma and social acceptability, cope with emotional and practical demands, and navigate structural and service-related constraints in rural epilepsy care.

## 2 Methods

### 2.1 Study design

This study employed a qualitative, exploratory-descriptive design to explore the lived experiences and challenges of acceptability among caregivers of PWE in Limpopo and Mpumalanga Provinces of South Africa. A qualitative approach was appropriate because the study sought to generate detailed accounts of caregivers’ day-to-day realities, meanings, and challenges within their social and health-system contexts. The exploratory-descriptive design allowed the researchers to examine participants’ experiences in their own words and to identify patterns across caregiver narratives using inductive thematic analysis. The reporting of this qualitative study was guided by the Consolidated Criteria for Reporting Qualitative Research (COREQ), which supports transparent reporting of qualitative research design, sampling, data collection, analysis, and researcher reflexivity (Tong et al., 2007).

### 2.2 Study setting

The study was conducted in rural and peri-urban communities in the Limpopo and Mpumalanga provinces of South Africa, including Collins Chabane Municipality in Vhembe District, Limpopo, and Bushbuckridge in Ehlanzeni District, Mpumalanga. These settings are characterized by poverty, limited health infrastructure, long distances to healthcare facilities, and strong traditional belief systems that may shape how epilepsy is understood and managed. These contextual factors made the study settings particularly relevant for understanding caregiving experiences, social acceptability, and access to support for families affected by epilepsy.

### 2.3 Study population and sampling

The study population consisted of informal caregivers of PWE. For this study, an informal caregiver was defined as an adult family member or relative who provided primary or substantial unpaid care to a person with a clinical diagnosis of epilepsy living in the same household. Participants included parents, grandparents, spouses, siblings, and other relatives involved in the day-to-day care of PWE.

A snowball sampling strategy was used to recruit participants. This approach was suitable because caregivers of PWE are not always readily identifiable through formal registers, particularly in rural communities where epilepsy may be hidden due to stigma, cultural misconceptions, or limited contact with formal services (Kaddumukasa et al., 2018; Kaddumukasa et al., 2019; Musekwa and Makhado, 2023). Initial participants were identified through community entry processes and local networks, and they subsequently referred other eligible caregivers. To reduce potential selection bias, recruitment was initiated through different community entry points and local networks across both provinces.

Participants were eligible for inclusion if they:

- were aged 18 years or older;
- were an informal caregiver of a person with a clinical diagnosis of epilepsy;
- lived in the same household as the person with epilepsy;
- had provided care for at least 6 months; and
- were willing and able to provide informed consent.

Caregivers were excluded if they were unable to provide informed consent, had limited direct involvement in caregiving, or were paid/formal care workers rather than informal family caregivers.

A total of 60 caregivers were interviewed, comprising 30 from Limpopo and 30 from Mpumalanga. In Mpumalanga, the sample comprised 10 males and 20 females, while in Limpopo, 4 males and 26 females participated. All participants were aged 18 years or older. The duration of caregiving was categorized as 6 months to 1 year, 1–3 years, and more than 3 years, with most participants having provided care for more than 3 years. Participant demographic characteristics are presented in Table 1.

**Table 1:** Participant demographic characteristics.

Characteristic	Mpumalanga	Limpopo	Total
Number of participants	30	30	60
Gender			
Male	10	4	14
Female	20	26	46
Age	≥18 years	≥18 years	≥18 years
Caregiver inclusion criterion	Minimum 6 months of caregiving experience	Minimum 6 months of caregiving experience	—
Predominant caregiving duration	Majority >3 years	Majority >3 years	Majority >3 years

Data saturation was monitored throughout data collection. After each interview, the researcher transcribed and reviewed the responses to identify new ideas, patterns, and themes related to caregivers’ experiences and the challenges of acceptability. Interviews continued until no new important information or themes emerged. Saturation was considered reached when three consecutive interviews yielded no new themes or information. The sample size was therefore guided by the richness and repetition of information rather than by a fixed numerical target.

### 2.4 Data collection

Data were collected through in-depth semi-structured interviews conducted between December 2025 and February 2026. Semi-structured interviews were chosen to allow participants to narrate their experiences freely while ensuring that all interviews covered key topics relevant to the study aim. All participants were asked the same core questions to ensure consistency and minimize interviewer bias. The interview guide explored key areas, including caregiving experiences, emotional and physical burden, social and cultural perceptions of epilepsy, challenges encountered in caregiving, experiences of stigma and discrimination, coping strategies, available support systems, and community attitudes towards PWE and their families. Probing questions were used where necessary to clarify responses and allow participants to elaborate on their experiences. This approach ensured both consistency in data collection and flexibility for in-depth exploration of participants’ perspectives.

Interviews were conducted in participants’ preferred languages, namely, Xitsonga, Tshivenda, Sepedi, siSwati, by the researcher H. N. in locations convenient and private for participants, such as their homes or another mutually agreed safe setting. Each interview lasted approximately 40–65 min. With participants’ consent, interviews were audio-recorded and supplemented by field notes documenting non-verbal expressions, contextual observations, and immediate analytic reflections.

Audio-recordings were transcribed verbatim. Where interviews were conducted in local languages, transcripts were translated into English for analysis by language experts. To preserve meaning and enhance accuracy, translations were checked against the original recordings and reviewed by H. N., L. M. and T. G. M.

### 2.5 Data analysis

Data were analyzed using inductive reflexive thematic analysis, following Braun and Clarke’s six-phase approach (Braun and Clarke, 2006; Braun and Clarke, 2019). Thematic analysis was selected because it enabled systematic identification, organization, and interpretation of patterns across caregiver accounts while remaining grounded in participants’ own descriptions. The analysis followed six iterative phases: familiarization with the data, generation of initial codes, development of candidate themes, review of themes against the coded extracts and full dataset, definition and naming of themes, and production of the analytic narrative.

The researchers first read and re-read the transcripts alongside field notes to become familiar with the data. Initial codes were then generated from meaningful segments of text relevant to the study aim. Related codes were grouped into preliminary categories and candidate themes. These themes were reviewed and refined by comparing them against coded extracts and the full dataset. Final themes were then defined and named to ensure that each captured a distinct aspect of caregivers’ experiences. Representative verbatim quotations were selected to illustrate each theme.

Coding and theme development were managed using ATLAS. TI Version 8.4, which supported data organization and analytic transparency. To enhance consistency, the authors discussed coding decisions and emerging themes with the independent co-coder until agreement was reached. Analysis was conducted across the pooled dataset from both provinces, with attention to areas of similarity and difference in emphasis between provinces.

Although coding was primarily inductive, interpretation of the final themes was sensitized by the transactional stress and coping model, the caregiving stress process model, and a psychosocial adaptation perspective, allowing the researchers to examine how caregivers appraised epilepsy-related demands, mobilized coping resources, and adapted under social and structural constraints (Lazarus and Folkman, 1984; Pearlin et al., 1990).

#### 2.5.1 Trustworthiness

Trustworthiness was ensured using the criteria of credibility, dependability, confirmability, and transferability. Credibility was enhanced through prolonged engagement with the data, the use of verbatim quotations, and regular discussions among the research team during analysis. Where applicable, member checking was undertaken by clarifying key issues during interviews and/or sharing summaries of emerging interpretations with selected participants.

Dependability was supported through a clear audit trail documenting the research process, including sampling decisions, interview procedures, coding development, and theme refinement. Confirmability was strengthened through reflexive note-taking and team discussions, which helped the researchers examine how their professional backgrounds and assumptions might influence their interpretations. Transferability was addressed by providing a detailed description of the study setting, participant characteristics, and caregiving context, enabling readers to assess the applicability of the findings to similar settings.

### 2.6 Ethical considerations

Ethical approval was obtained from the University of Venda Human and Clinical Trials Research Ethics Committee (HCTREC) (Ethical Clearance No. FHS/25/PH/22/1811). Permission to conduct the study was also obtained from relevant traditional authorities and community leaders in the participating communities. Participation was voluntary, and written informed consent was obtained from all participants before data collection. Participants were informed about the purpose of the study, the voluntary nature of participation, their right to decline to answer any question, and their right to withdraw from the study at any time without penalty. Confidentiality and anonymity were maintained by removing identifying information from transcripts and using pseudonyms or participant codes in reporting. Audio files, transcripts, and field notes were stored securely and were accessible only to the research team.

## 3 Results

Analysis of caregivers’ accounts across Limpopo and Mpumalanga revealed five interrelated themes: (1) caregiving as constant vigilance, (2) emotional and physical strain in responding to unpredictable seizures, (3) stigma and low social acceptability of epilepsy, (4) caregiving under structural and service constraints, and (5) coping, endurance, and unmet support needs. Across both provinces, caregiving was described as an ongoing and demanding responsibility shaped by the unpredictability of seizures, social misunderstanding of epilepsy, and limited practical support. Although caregiving experiences were broadly similar across Limpopo and Mpumalanga, some differences were observed in the emphasis participants placed on supervision burden, stigma experiences, emotional distress, and access to support services.

### 3.1 Theme 1: Caregiving as constant vigilance

Caregivers in both provinces described epilepsy care as requiring uninterrupted watchfulness. Because seizures were perceived as sudden, dangerous, and difficult to predict, participants felt they could not safely leave the person with epilepsy alone, even for short periods. Daily life became organized around monitoring, anticipating triggers, and reducing exposure to danger.

A caregiver from Limpopo described this constant state of alertness as follows:

*“The fact that I always have to be with the person with epilepsy 24/7. I can’t even go to the store, I can’t even do anything”* (Participant 15, Limpopo).

Another Limpopo participant explained how uncertainty about when a seizure might happen shaped everyday life:

*“I feel like every day is a typical day who is responsible for a child with epilepsy because you don’t know what to expect, you don’t know when it can happen and you just need to be ready at all times or anything that can happen so it is always tough you don’t know when it is going to happen and the reaction and if you will be in public or not so yeah it is quite hectic”* (Participant 26, Limpopo).

Participants in Mpumalanga conveyed the same sense of relentless surveillance. One participant stated:

*“You can’t even take an hour without seeing them… You have to make sure you are always here with them… So it is difficult sometimes because I want to go out, and I can’t… Because I can’t leave him”* (Participant 3, Mpumalanga).

Another caregiver highlighted how monitoring extended even to highly private and routine tasks:

*“You must understand that when a person has epilepsy, that person is no longer normal… in everything, you must always be there for them, especially when it comes to them using the pit toilet alone. That is what makes me worried. Because I have to accompany him to the toilet until they finish to make sure they are safe”* (Participant 3, Mpumalanga).

This vigilance was not limited to physical presence. It also involved active safety management, such as avoiding triggers, preventing access to fire, and carefully structuring the environment. A Limpopo caregiver explained:

*“In short, I would say that… living with epilepsy, you must be alert all the time. People are triggered by different things; some might be dams or water, while others are triggered by fire. So when caring for someone with epilepsy, you need to be more cautious”* (Participant 14, Limpopo).

Similarly, a participant from Mpumalanga said:

*“The most demanding parts are ensuring that they don’t fall near dangerous places, like around fire or sharp objects. I have to ensure safety”* (Participant 14, Mpumalanga).

Taken together, these accounts show that caregiving was experienced not as occasional assistance during seizures, but as constant vigilance that reorganized time, movement, domestic routines, and personal freedom.

### 3.2 Theme 2: Emotional and physical strain in responding to unpredictable seizures

The unpredictability of seizures generated intense emotional distress. Caregivers repeatedly described fear, panic, helplessness, and shock, especially when seizures involved collapse, loss of consciousness, tongue-biting, foaming, incontinence, or prolonged recovery. In many cases, the emotional strain was intensified by limited knowledge of seizure first aid, leaving caregivers uncertain about what to do in the moment.

One caregiver from Limpopo gave a vivid account of being alone during a seizure:

*“In my family we have someone who is epileptic… till a day it was me and him in the house and he started experiencing seizures and all the symptoms of epilepsy I was shocked and I didn’t know what to do but with the little knowledge of epilepsy that I had I know that the first thing is to put something in their mouth between their teeth so that the person doesn’t not bite themselves so that is what I did and from then I was so scared I didn’t know what to do. I just called someone who used to care for him, and called the emergency care. It was very difficult for me to experience this because I did not know what to do”* (Participant 16, Limpopo).

Another Limpopo participant reflected on the ongoing uncertainty of caring for a child with epilepsy:

*“The biggest challenge that I face when I am caring for this child is that I always don’t know what is going to happen tomorrow because I feel like the episode can happen now but I’m not sure if it is going to stop or I am losing my child, or not so that is the biggest challenge”* (Participant 26, Limpopo).

Mpumalanga caregivers described similarly traumatic episodes. One participant recounted:

*“It was a very difficult day because it was my 1st time seeing a person with epilepsy doing that. He started falling, being stiff, biting his teeth, and he peed on himself. I was so shocked, and I tried to put my fingers between his teeth, but he started biting me”* (Participant 19, Mpumalanga).

Another said:

*“When the seizures begin, I panic, and I end up trying to help. When I see foam coming out of their mouths, I think they are drying out. When they bite their tongue, I even try to put my hand in their mouth or try to pour water to try to stop the seizures. I don’t have much knowledge on how to respond”* (Participant 20, Mpumalanga).

Alongside emotional distress, caregivers also described the physical burden of care, especially when they had to lift or bathe the person with epilepsy or assist after falls. This was particularly difficult for older caregivers or those already living with health problems. A Limpopo participant explained:

*“The biggest challenge is when I am left alone with her, and she has an episode. It is a challenge because I don’t have enough strength to lift her, as we are not the same size. When she falls, I have to hurry and get people to help. If I don’t hurry up, then I always get scared that she might die”* (Participant 21, Limpopo).

A caregiver from Mpumalanga similarly described the physical demands in stark detail:

*“Yooo, this is one of bathing her… she is 13, and she’s just nearly as big as I am. I’m old and 72 years old, but I weigh 68 kg. So just imagine bathing her, putting her clothes on”* (Participant 5, Mpumalanga).

These accounts show that the strain of caregiving was both emotional and embodied. Fear of death or injury, combined with the physical labor of responding to seizures and supporting daily activities, made caregiving deeply exhausting.

### 3.3 Theme 3: Stigma and low social acceptability of epilepsy

Caregiving took place within a context where epilepsy was often poorly understood and socially devalued. Participants described communities in which PWE were judged, mocked, avoided, or treated as socially inferior. This stigma extended beyond the person with epilepsy to affect the caregiver and the wider family, creating what many participants experienced as shame, hurt, and social withdrawal.

In Limpopo, caregivers described how exclusion operated in schools, families, and neighborhoods. One participant said:

*“They don’t treat us well, they talk badly about us like we are nothing, even when she approaches them, they run as if they see a ghost. Even young children are told not to talk to her, they get scared”* (Participant 12, Limpopo).

Another participant explained how stigma followed the family into social spaces:

*“It affects us because even when we have to go out, people gossip about us, so it hurts, even at school, they mock us because our grandma has an illness, they call us names”* (Participant 12, Limpopo).

Participants also described how epilepsy was interpreted through culturally loaded ideas of curse, witchcraft, madness, or spiritual possession. A Limpopo caregiver stated:

*“In general, they view epilepsy as something that has to do with rituals; they think it has to do with demons or witchcraft… so they always view it like there is African magic happening there”* (Participant 26, Limpopo).

Another said:

*“Some say he is performing witchcraft. Some say he has a spiritual gift because of how his body moves when he has seizures”* (Participant 11, Limpopo).

Mpumalanga caregivers echoed these experiences strongly. One participant said:

*“People don’t understand epilepsy. Some think the patient is demon-possessed and that they are bewitched. They will tell you to go to a Sangoma [Traditional healer] for traditional medicine”* (Participant 23, Mpumalanga).

Another described the painful experience of rejection in church:

*“It hurts! It hurts! I don’t want to lie, it hurts! Like when I overheard the lady at church say, “You do not know that she is crazy; we know she is crazy…“That’s why when I’m at church, I avoid standing with other people… But I was just hurt and avoiding going to church”* (Participant 5, Mpumalanga).

School exclusion was also prominent in Mpumalanga. One caregiver explained:

*“It was a very big challenge because he experienced discrimination even at school. His friends isolated him. And he had such low self-esteem and depression, to the point that he was suicidal. We were affected very badly as a family, to the point that we had to take him out of that school for the sake of his mental health”* (Participant 18, Mpumalanga).

These narratives show that caregiving burdens were intensified by the low social acceptability of epilepsy. Caregivers were not only managing seizures and safety, but also navigating gossip, social rejection, and culturally rooted misconceptions that undermined dignity and belonging.

### 3.4 Theme 4: Caregiving under structural and service constraints

Caregivers’ experiences were also shaped by structural hardship, including poverty, disrupted livelihoods, transport costs, and limited access to supportive health and community services. Many participants described having to sacrifice work, school, or income-generating activities because caregiving could not be postponed or delegated safely. A caregiver from Limpopo explained how care responsibilities constrained employment:

*“The biggest challenge that I face it is when I have to go somewhere or get a job somewhere that is far… I have to find a job that I can go and come back the same day… because if I get a job in Johannesburg, I can’t accept it because I don’t know how I’m going to survive”* (Participant 24, Limpopo).

Another highlighted the financial demands of care:

*“Taking her to the hospital requires money transport is required… There are certain food items that she should eat… I have to [pay] the person who takes care of her when I’m not around, so money is needed all the time”* (Participant 21, Limpopo).

In Mpumalanga, caregivers likewise described loss of work and educational opportunities. One participant said:

*“I had to stop working because you can get called at random, people telling you that your child is having seizures. Right now we don’t have an income nor grant”* (Participant 25, Mpumalanga).

Another elaborated on how caregiving displaced both employment and further study:

*“I had to stop working. I also started a short course with a higher institution, and I had to stop that and concentrate on caregiving for my child with epilepsy. He can have seizures at any time, when I’m cooking, studying”* (Participant 25, Mpumalanga).

Caregivers also described gaps in formal support services. In Limpopo, one participant stated plainly:

*“I can say they don’t care because no one comes and registers us… I have never seen home-based care people visit us”* (Participant 1, Limpopo).

In Mpumalanga, service gaps were often experienced alongside isolation and lack of practical help:

*“To be honest, I’m not coping. As I have said, I don’t have any support. As a result, I can’t have a social life because I will worry about their safety if I go out. What if I go out and they have seizures and hit something dangerous and die? So, I must always make sure that I’m home”* (Participant 20, Mpumalanga).

These findings suggest that caregiver burden was not produced by epilepsy alone, but by the intersection of caregiving demands with poverty, service limitations, transport barriers, and the absence of reliable respite or home-based support.

### 3.5 Theme 5: coping, endurance, and unmet support needs

Despite the intensity of caregiving, participants described ways to cope and to sustain care over time. This included persistence, acceptance, reliance on treatment, counselling from healthcare workers, family assistance, and practical strategies for monitoring medication and triggers. At the same time, many caregivers stressed that these coping mechanisms were fragile and insufficient without stronger formal and community support.

In Limpopo, several caregivers identified treatment and clinic support as important sources of hope. One participant said:

*“The support we have is from health practitioners at the hospital”* (Participant 2, Limpopo).

Another explained:

*“As a parent, I try by all means not to let it affect me… whenever I go to the clinic, I explain to them what I am facing, and they help me with counseling, and they give me good advice”* (Participant 13, Limpopo).

Caregivers also described practical self-management strategies. For example:

*“I handle them by setting a reminder for myself and make sure that I avoid such incidents like skipping giving the child medication… and also you need to give medication at this time”* (Participant 26, Limpopo).

In Mpumalanga, endurance was often framed in terms of responsibility and emotional survival. One participant said:

*“It’s very demanding, and sometimes I break down. But when I look at the next person I know, I have to be strong since they can’t take care of themselves. It’s my responsibility”* (Participant 20, Mpumalanga).

Another caregiver emphasized the need for patience and emotional restraint:

*“okay my typical day as a caregiver, you have to be careful and not be harsh, but be welcoming”* (Participant 5, Mpumalanga).

Yet these coping efforts coexisted with clear unmet needs. Some participants wanted more information about epilepsy, stronger community education, practical seizure-response training, and better social support. A participant from Mpumalanga captured this gap succinctly:

*“It is not understood because it is a rare medical condition, and we don’t have enough information about it. We never had education about the disease, so it became a taboo. The community hasn’t accepted epilepsy well”* (Participant 14, Mpumalanga).

Similarly, one Limpopo caregiver pointed to the limits of family endurance in the absence of resources:

*“Money, not having money, is stressful because talking to someone and not having money doesn’t help with anything”* (Participant 21, Limpopo).

Caregivers’ accounts show that coping was shaped less by the absence of distress than by the determination to continue caring despite fear, stigma, poverty, and uncertainty. Their narratives also point to the need for more structured psychosocial, educational, and community-based support.

## 4 Discussion

Interpreted through stress-coping and psychosocial adaptation perspectives, the findings show that epilepsy caregiving in rural Limpopo and Mpumalanga is best understood as a process of constrained psychosocial adaptation. Caregivers appraised seizure unpredictability, risk of injury, physical care, stigma, and financial strain as persistent stressors rather than isolated caregiving events (Lazarus and Folkman, 1984). Within the caregiving stress process model, seizure management and safety concerns can be understood as primary stressors, while disrupted work, reduced mobility, social exclusion, transport costs, and financial insecurity represent secondary role strains (Pearlin et al., 1990). Caregivers responded through constant vigilance, treatment monitoring, faith, patience, acceptance, and selective use of health services. However, these coping responses were constrained by poverty, transport barriers, limited respite care, inadequate caregiver education, and low social acceptability of epilepsy.

This interpretation extends existing epilepsy caregiving literature by showing that caregiver burden in these rural South African settings is not produced by seizure management alone. Rather, it emerges through the interaction of clinical uncertainty, emotional labour, affiliate stigma, social exclusion, and weak service support (Hansen et al., 2018; Keikelame and Swartz, 2016; Sabo et al., 2020; Yu et al., 2022). Coping was therefore not simply an individual strength but a fragile, socially shaped response to caregiving demands under conditions of structural constraint.

A key finding was that caregiving was characterized by constant vigilance. Caregivers described the need to remain alert, physically present, and continuously assess risk in both domestic and community environments. Daily activities such as working, shopping, bathing, or resting were reorganized around the possibility of sudden seizures. This supports earlier research showing that seizure unpredictability disrupts routines and increases role strain (Keikelame and Swartz, 2016; Gordon et al., 2012). Importantly, the present study extends this understanding by showing that vigilance is not only emotional but also spatial and practical, as caregivers continuously evaluated environmental risks such as roads, fires, water, and toilets. Caregiving, therefore, functioned as ongoing risk management rather than episodic support.

The findings also highlighted substantial emotional and physical strain during seizure episodes. Caregivers reported fear, panic, helplessness, and shock, particularly during severe seizures involving collapse, injury, or loss of consciousness. Limited knowledge of seizure first aid sometimes led to unsafe responses, reflecting evidence that inadequate training increases distress and affects care practices (Kaddumukasa et al., 2019; Musekwa and Makhado, 2023). Emotional burden was closely linked to fear of harm or death and the sense of sole responsibility during episodes (Cui et al., 2025; Poprelka et al., 2025). Physically, caregivers often had to lift individuals after falls, assist with bathing and toileting, and provide care despite their own health or age-related limitations. This reflects the embodied nature of epilepsy caregiving, particularly in low-resource rural settings (Sabo et al., 2020).

A central finding was the pervasive role of stigma and the low social acceptability of epilepsy. Caregivers described widespread misconceptions linking epilepsy to witchcraft, curses, madness, or contagion, which shaped exclusionary attitudes in schools, churches, and communities. These findings are consistent with African literature documenting persistent epilepsy-related stigma and cultural misconceptions (Agbetou et al., 2023; Kaddumukasa et al., 2018; Nemathaga et al., 2023). Importantly, stigma extended beyond PWE to caregivers, who experienced affiliate stigma, including gossip, judgment, and social exclusion (Hansen et al., 2018). This demonstrates that acceptability is not only about the person with epilepsy but also about how families are socially positioned within their communities.

Caregiving also occurred within significant structural and service constraints. Participants described poverty, unemployment, transport barriers, and loss of income opportunities due to caregiving demands. These challenges reflect broader systemic inequalities and gaps in rural healthcare access (Ngene et al., 2023; Wagner et al., 2020). The findings show that caregiver burden is shaped not only by individual or family circumstances but also by weak service infrastructure, limited epilepsy-specific support, and lack of respite care. This reinforces the need to frame epilepsy caregiving as a public health and systems issue rather than a private family burden (WHO, 2019).

Despite these challenges, caregivers demonstrated coping, endurance, and adaptive strategies. These included reliance on faith, family responsibility, patience, treatment adherence, and informal monitoring of triggers and medication schedules. However, these strategies were often fragile and insufficient. Participants expressed clear needs for epilepsy education, seizure-response training, psychosocial support, and improved access to services. This aligns with evidence showing that lack of training and support perpetuates uncertainty and reliance on informal practices (Musekwa et al., 2024; Nemathaga et al., 2025). While resilience was evident, it should not obscure the substantial unmet support needs and ongoing burden experienced by caregivers.

Although caregivers in both provinces described epilepsy caregiving as demanding, the findings also revealed differences in emphasis between Limpopo and Mpumalanga. In Limpopo, participants more frequently foregrounded constant supervision, transport difficulties, financial strain, and limited access to home-based or community support services. Stigma was also strongly linked to cultural interpretations of epilepsy, including beliefs about witchcraft, rituals, or spiritual causes. These findings are consistent with South African and wider sub-Saharan African literature showing that epilepsy is often interpreted through cultural and spiritual explanatory models, which may influence stigma, help-seeking, and treatment pathways (Kaddumukasa et al., 2018; Makhado et al., 2024; Nemathaga et al., 2023). In Mpumalanga, participants placed stronger emphasis on emotional exhaustion, school-based discrimination, church-related rejection, and the absence of practical social support. These accounts reflect evidence that epilepsy-related stigma affects not only PWE but also family members and caregivers through affiliate stigma, social withdrawal, and increased caregiver burden (Agbetou et al., 2023; Hansen et al., 2018; Shi et al., 2021). Despite these differences, caregivers across both provinces shared experiences of seizure unpredictability, fear of injury or death, disrupted work and household routines, and reliance on fragile coping strategies. This suggests that epilepsy caregiving in both settings is shaped by common rural constraints, while the specific manifestations of stigma and support gaps vary across local social and service contexts (Ngene et al., 2023; Wagner et al., 2020).

Taken together, the findings show that acceptability is central to the caregiving experience. It operates at social, interpersonal, and household levels, shaping whether epilepsy is understood, whether PWE are included in community life, and whether caregiving demands are tolerated within families. Low acceptability intensified stigma, isolation, and caregiver distress, highlighting that social responses are an integral part of the caregiving environment. Improving outcomes, therefore, requires interventions that extend beyond clinical care to include community education, stigma reduction, caregiver training, and psychosocial support at the primary healthcare level.

Although experiences across Limpopo and Mpumalanga were broadly similar, some contextual differences emerged. Mpumalanga narratives more strongly emphasized fear of injury during seizures and gaps in formal and community support, while Limpopo accounts more often highlighted stigma linked to cultural beliefs and misunderstanding of epilepsy. However, caregivers in both provinces shared similar coping strategies, including reliance on family, faith-based coping, and practical caregiving adaptations. These findings suggest that while epilepsy-related stigma is a shared challenge, its expression is shaped by local socio-cultural and health system contexts (Willie, 2025; Keikelame and Swartz, 2016; Hohmann et al., 2022). This study contributes to the literature by providing a contextualized account of epilepsy caregiving in two under-researched rural provinces in South Africa. It shows that caregiving is shaped by continuous vigilance, embodied labor, stigma, structural inequality, and limited support systems. Caregivers remain central to epilepsy care, yet are insufficiently supported in policy and practice. A more effective response requires integrated interventions that address clinical care, caregiver education, psychosocial support, and community-level stigma reduction. Caregiving for PWE in these settings is a demanding and often isolating responsibility, extending far beyond seizure management and requiring sustained attention to the social and structural realities of rural life.

## 5 Implications

This study presents significant implications for policy, practice, and research concerning epilepsy care in rural South Africa. Firstly, the findings illustrate that caregiver burden extends beyond mere seizure management; it is intricately linked to continuous vigilance, emotional strain, social stigma, and structural hardships. Consequently, policy initiatives should transcend a narrow biomedical perspective and acknowledge caregivers as pivotal figures in epilepsy care who necessitate comprehensive practical, psychosocial, and economic support. It is imperative to enhance epilepsy care at the primary healthcare level, ensure consistent access to antiseizure medications, and mitigate transport-related barriers, particularly in rural and under-resourced contexts.

Secondly, these findings bear important ramifications for clinical practice. Frontline healthcare workers, especially those involved in primary care and community-based services, must be better equipped to deliver caregiver-centered epilepsy education, counseling, and practical guidance on managing seizures. Caregivers within this study frequently expressed feelings of fear, uncertainty, and reliance on unsafe or myth-based first-aid practices, underscoring the urgent need for structured psychoeducation and practical training. By integrating caregiver counseling and support into routine epilepsy follow-ups, we can enhance caregiver confidence, alleviate distress, and improve the quality of care within home environments.

Thirdly, the study underscores the necessity for community-level interventions aimed at enhancing the social acceptability of epilepsy. Public education campaigns, school sensitization initiatives, and engagement with religious institutions, traditional leaders, and broader community stakeholders can help diminish misconceptions, judgment, and social exclusion. Given that stigma impacts both individuals with epilepsy and their caregivers, anti-stigma efforts should adopt a comprehensive approach that targets families and communities rather than focusing solely on individuals.

Finally, the findings identify several priorities for future research. Longitudinal studies are essential to delve into how caregiving burdens and coping mechanisms evolve over time, particularly in households grappling with chronic poverty and limited access to services. Additionally, further investigation is warranted into interventions such as caregiver support groups, home-based education programs, community awareness campaigns, and integrated clinic-community care models. Engaging individuals with epilepsy, educators, community leaders, and healthcare providers in future research endeavors will contribute to a more nuanced understanding of how epilepsy acceptability is shaped across diverse social contexts.

## 6 Strengths

This study has several strengths. It provides a rich qualitative account of caregiving for PWE across two under-researched rural South African provinces, allowing both cross-site comparison and contextual depth. The use of in-depth interviews enabled caregivers to describe their experiences in their own words, while the inclusion of participants from Limpopo and Mpumalanga broadened the relevance of the findings within rural South African settings. Conducting interviews in participants’ preferred languages also enhanced data accessibility and authenticity. In addition, the use of inductive thematic analysis supported a grounded interpretation of caregiver experiences and allowed key patterns to emerge directly from participant narratives. The study’s focus on both lived experience and acceptability challenges further strengthens its contribution by showing how caregiving is shaped by seizures themselves, stigma, social responses, and structural context.

## 7 Limitations

This study should be interpreted in light of several limitations. The use of snowball sampling may have introduced selection bias. Caregivers who were more socially connected, more visible in the community, or more willing to discuss epilepsy may have been more likely to participate, while isolated caregivers, caregivers outside referral networks, or those experiencing severe stigma may have been underrepresented. This may have shaped the range of experiences captured, particularly regarding stigma, social support, service access, and coping. Although recruitment across two provinces and different community networks improved variation, the findings remain context-specific and should not be interpreted as statistically generalizable. This limitation is important because epilepsy-related stigma may influence disclosure, help-seeking, and willingness to participate in research (Kaddumukasa et al., 2018; Hansen et al., 2018; Alege et al., 2026; Shi et al., 2021). In addition, because interviews were conducted and translated across multiple languages, some nuances of meaning may have been lost despite careful transcription and review. The qualitative design captured experiences at a single point in time and could not examine how caregiving challenges or coping strategies changed over time. Finally, as with all thematic analysis, interpretation was shaped by the researcher’s judgment, although efforts to enhance rigor through reflexive engagement, team discussion, and an audit trail helped strengthen analytic credibility. The study did not collect information regarding participants’ prior exposure to anti-stigma or epilepsy awareness interventions. This may have influenced participants’ perceptions and experiences of stigma and should be considered in future studies.

## 8 Future recommendations

This study highlights the need for a multifaceted approach to improve the acceptability and caregiving experiences of PWE. At the community level, targeted educational campaigns involving stakeholders such as teachers, traditional and religious leaders, and healthcare providers are needed to address misconceptions, reduce stigma, and promote social inclusion. Early, school-based interventions are particularly important to foster understanding of epilepsy from a young age.

At the healthcare level, strengthening training and capacity among healthcare providers, especially in rural settings, is essential to improve epilepsy management, patient education, and caregiver support. Efforts should also focus on improving access to care by addressing barriers to availability, affordability, and treatment continuity.

At the policy and systems level, coordinated efforts from government and healthcare systems are needed to develop and implement comprehensive, community-based programs that integrate medical, psychosocial, and educational support for PWE and their families.

From a research perspective, future qualitative studies should expand participation to include a broader range of stakeholders and consider comparative or multi-site designs across provinces and neighboring countries. Participatory approaches involving caregivers and PWE are recommended to ensure that interventions are contextually relevant and responsive to lived experiences. Additionally, further research is needed to explore factors influencing treatment adherence, cultural beliefs, and social determinants that shape the acceptability of epilepsy.

## 9 Conclusion

Caregiving for PWE in Limpopo and Mpumalanga emerged as a demanding, continuous, and socially shaped responsibility. Caregivers described lives organized around vigilance, fear of unpredictable seizures, physical effort, and the need to protect the person with epilepsy from harm. At the same time, they had to navigate community stigma, cultural misconceptions, social exclusion, and weak support systems. These burdens were intensified by poverty, disrupted livelihoods, transport barriers, and limited access to practical and psychosocial support. Although caregivers demonstrated resilience through endurance, treatment monitoring, faith, and a strong sense of family responsibility, these coping strategies were often insufficient in the face of ongoing emotional strain and structural disadvantage. The findings, therefore, suggest that improving epilepsy outcomes in rural South Africa requires more than seizure control alone. It requires family-centered and community-responsive interventions that support caregivers, strengthen public understanding of epilepsy, reduce stigma, and improve access to practical guidance and health services. This study highlights that caregivers are central to epilepsy care, yet they remain insufficiently recognized and supported. A more effective response to epilepsy in rural South Africa should combine caregiver education, anti-stigma strategies, stronger primary healthcare support, and community-based mechanisms that reduce the social and structural burden of care.

## Data Availability

The raw data supporting the conclusions of this article will be made available by the authors, without undue reservation.
